# Visual Difficulties and Uptake of Optical Correction Among Malawians: Insights from the 2024 Demographic and Health Survey

**DOI:** 10.3390/ijerph23070841

**Published:** 2026-06-26

**Authors:** Joan Efua Edua Hanson, Selassie Tagoh, Ernest Korang Asamoah, Susarah Maria Richter, Michael Agyemang Kwarteng

**Affiliations:** 1 Ophthalmology Department, University Hospital Coventry and Warwickshire, Coventry CV2 2DX, UK; j.hanson6@nhs.net; 2 Department of Medicine, Dunedin School of Medicine, University of Otago, Dunedin 9054, New Zealand; sellsell488@gmail.com; 3 Department of Optometry, Faculty of Science, Bindura University of Science Education, Bindura Private Bag 1020, Zimbabwe; 4 Centre for Health and Social Care, Institute for Educational Research, Global Banking School, Birmingham B1 2JB, UK; easamoah@globalbanking.ac.uk; 5 Department of Optometry, Faculty of Health Sciences, University of Johannesburg, Johannesburg 2006, South Africa; 6 Optometry Unit, Department of Clinical Surgical Sciences, Faculty of Medical Sciences, The University of the West Indies, St. Augustine 685509, Trinidad and Tobago; 7 Centre for Vision across the Life Span, Department of Optometry and Vision Science, School of Applied Sciences, University of Huddersfield, Huddersfield HD1 3DH, UK

**Keywords:** visual impairment, refractive error, optical correction, Malawi, health equity

## Abstract

**Highlights:**

**Public health relevance—How does this work relate to a public health issue?**

- Visual difficulty and uncorrected refractive error are major yet preventable public health challenges.
- Using nationally representative 2024 Malawi Demographic and Health Survey data, this study examines visual difficulty and optical correction uptake across population groups in Malawi.

**Public health significance—Why is this work of significance to public health?**

- The study identifies a large unmet need for visual difficulties, with many affected individuals not using spectacles or contact lenses.
- Older adults, rural residents, widowed individuals, and those with lower education experienced greater burden, highlighting inequities in eye health.

**Public health implications—What are the key implications or messages for practitioners, policy makers and/or researchers in public health?**

- Expanding affordable refractive services in rural and underserved areas is critical to reducing avoidable visual impairment.
- Community screening, eye health education, and equitable eye care policies are needed to improve correction uptake and reduce disparities.

**Abstract:**

Background: Uncorrected visual impairment, primarily due to refractive errors, is a persistent public health concern in low- and middle-income countries. In Malawi, national data on the prevalence of visual difficulty and uptake of optical correction remain scarce, hindering targeted health interventions. This study examines the prevalence of self-reported visual difficulties, the uptake of refractive correction, and the sociodemographic determinants of optical correction in Malawi using 2024 national survey data. Methods: We conducted a secondary analysis of data from the 2024 Malawi Demographic and Health Survey (MDHS), a nationally representative, cross-sectional survey encompassing 23,095 participants across urban and rural regions. Self-reported visual difficulties and optical correction (spectacle/contact lens use) were assessed alongside key sociodemographic factors. Associations were examined using chi-square tests and multinomial logistic regression. Results: Among 23,095 participants, visual difficulty was significantly associated with age, education, marital status, region, and residence (all *p* < 0.001). The prevalence of visual difficulty increased markedly with age, from 2.3% in the youngest group to 15.5% in those over 74 years. Women, individuals with lower education, widowed participants, and residents of the Northern region experienced higher rates of visual difficulty. Spectacle or contact lens use was low overall (6.4%), with uptake increasing with age, education, and urban residence but remaining below 20% even among the most educated. Most individuals reporting visual difficulty were not using optical correction, including 70% of those with “some difficulty” and 67% of those with “a lot of difficulty” indicating substantial unmet need. Multivariable analysis identified advancing age, higher education, urban residence, and Northern region as key independent predictors of both visual difficulty and refractive correction uptake. Conclusions: The unmet need for refractive correction in Malawi is substantial, disproportionately affecting older, less educated, rural, and widowed individuals. Expanding access to affordable and equitable refractive services is essential to reducing avoidable visual impairment and addressing social and geographic health disparities.

## 1. Introduction

Visual difficulty and vision impairment remain a major global public health concern, with substantial implications for health, education, economic productivity, and social inclusion. The World Health Organization (WHO) estimates that at least 2.2 billion people worldwide live with distance or near visual problems, of whom approximately one billion have conditions that could have been prevented or have yet to be addressed [1]. Uncorrected refractive errors remain one of the leading and most avoidable causes of visual difficulty globally, contributing disproportionately to vision loss in low-and middle-income countries (LMICs) [1,2]. Despite the availability of effective and low-cost interventions, significant inequities persist in access to basic vision care services.

Refractive errors, including myopia, hyperopia, astigmatism, and presbyopia, affect individuals across the life span and, when left uncorrected, can significantly hinder daily functioning which can lead to productivity loss and reduce quality of life [1,2]. Refractive correction, most commonly using spectacles, remains one of the most cost-effective health interventions available and is included in the WHO Priority Assistive Products List [3]. However, the existence of refractive need does not necessarily translate into utilization of corrective services. Consequently, the concept of uptake of refractive correction is increasingly recognized as a critical indicator of health system performance, encompassing not only service availability but also affordability, accessibility, awareness, and quality of care [4,5]. Taken together, these findings highlight that uncorrected refractive error (URE) is not only highly prevalent but also associated with substantial functional, educational, and economic consequences, particularly in LMICs where access to correction remains limited.

Evidence consistently demonstrates wide disparities in refractive correction uptake between and within countries. Recent global estimates of effective refractive error coverage (eREC) suggest that while coverage exceeds 80% in many high-income settings, it remains below 30% in sub-Saharan Africa (SSA) [5]. Structural barriers, including shortages of trained eye care personnel, uneven geographical distribution of services, out-of-pocket costs, and limited population awareness, continue to restrict access to refractive care in LMICs [2,6,7,8]. These barriers reinforce avoidable vision loss and perpetuate broader social and economic inequalities.

Malawi faces many of these systemic challenges. Although progress has been made in strengthening eye health services, access to refractive care remains constrained, particularly in rural and underserved areas [3,9]. Given the high burden of URE reported across SSA, it is likely that a substantial proportion of visual difficulty in Malawi is avoidable yet remains insufficiently addressed. Existing evidence on visual difficulty in Malawi is limited and largely derived from sub-national, facility-based, or age-specific studies, restricting generalizability to the national population [10]. Studies in similar settings have largely relied on such designs, which, while informative, are limited by selection bias, restricted generalizability, and therefore, may not adequately capture population-level patterns of visual difficulty or unmet need for refractive correction. Moreover, there is a notable absence of recent nationally representative data examining both the prevalence of visual difficulty and the extent to which individuals requiring correction can obtain and use refractive services. Such evidence is essential for informing equitable eye health policy, service planning, and progress towards universal health coverage.

Population-based surveys provide a critical platform for addressing these evidence gaps. The Demographic and Health Survey (DHS) program offers nationally representative data widely used to assess health outcomes, inequalities, and service utilization across LMICs [11]. The inclusion of vision-related questions in the 2024 Malawi Demographic and Health Survey presents a timely opportunity to examine visual difficulty and refractive correction uptake at scale, and to explore socio-demographic differentials associated with unmet need. Generating such evidence is particularly important in the context of global commitments to increase effective refractive error coverage by 2030 and reduce avoidable vision difficulty worldwide [5,12].

This study aims to examine visual difficulty and the uptake of refractive correction among Malawians by utilizing data from the 2024 Demographic and Health Survey. Specifically, the objectives are to estimate the prevalence of self-reported visual difficulty in Malawi, assess the proportion of individuals with visual difficulty who use refractive correction, and identify socio-demographic factors associated with the uptake of refractive correction.

## 2. Materials and Methods

### 2.1. Study Design and Setting

This study was a secondary analysis of cross-sectional survey data from the Malawi Demographic and Health Survey (MDHS) 2024, a nationally representative household survey conducted May–August 2024 [13]. The MDHS was carried out by the Malawi National Statistical Office (NSO) with support from the DHS Program [13]. It covered all regions of Malawi and was designed to yield representative estimates at the national level, for urban and rural areas, and for each district and city [13].

### 2.2. Sampling and Sample Size

The MDHS used a multistage stratified cluster design: 792 primary sampling units (clusters) were selected from the 2018 census frame, and approximately 23,070 households were targeted for interview [13]. In the end, 22,414 occupied households were successfully interviewed (a 99% response rate) [13]. All usual residents in these households were listed, and individuals of all ages provided data on disability and vision [13]. As this study is based on secondary analysis of nationally representative survey data, no a priori sample size calculation was undertaken for the present analysis. Our analytic sample comprised 23,095 individuals with complete data on vision and refractive error variables from the MDHS dataset. This large, national representative sample provides sufficient statistical power to estimate prevalence and examine associations across socio-demographic subgroups in this study.

### 2.3. Variables

The primary outcomes were (1) self-reported visual difficulty, categorized as “no difficulty,” “some difficulty,” “a lot of difficulty,” “cannot see at all,” or “don’t know,” and (2) wearing spectacles or contact lenses (yes/no). We defined these based on the DHS disability module questions on seeing ability. Covariates included age, sex, region, urban/rural residence, education, and marital status.

### 2.4. Data Analysis

All analyses were conducted using SPSS version 31 (IBM Corp., Armonk, NY, USA). Descriptive statistics (frequencies and percentages) were used to summarize the demographic characteristics of the study population. Associations between demographic variables and both visual difficulty and spectacle/contact lens use were examined using Chi-square tests. Multinomial logistic regression was performed to identify demographic predictors of visual difficulty and refractive correction uptake, allowing comparison of multiple outcome categories relative to a reference group while adjusting for potential confounders. Results are reported as odds ratios (ORs) and 95% confidence intervals (CIs). Statistical significance was set at *p* < 0.05.

## 3. Results

### 3.1. Demographic Distribution of Individuals Surveyed

A total of 23,095 participants were included in the analysis (Table 1). Most participants were aged 20–44 years, with peak representation in the 25–39 age group (mean 12.5%). The sample was predominantly male (62.3%), rural (78.7%), and from Malawi’s Southern region (45.7%). Over half of them had primary education (55.7%) while only 5.6% had higher education, and 1.3% did not know their education level. The majority were married or living with a partner (68.5%).

### 3.2. Association Between Demographic Factors and Visual Difficulty Among the Population Surveyed

Results of the Chi-square test (Table 2) showed a statistically significant association between all demographic factors and the report of visual difficulty (*p* < 0.001). Notably, the proportion of participants with “some visual difficulty” increased with age (2.3% at younger ages and 15.5% in the > 74-year-olds). A similar trend is observed for participants who had “a lot of difficulty” with vision (0% at younger ages and 8.7% in those older than 74 years). Those with no visual difficulty decreased from 96.0% (15–19 years) to 56.8% (>74 years).

Although the number of males who had some form of visual difficulty (“some difficulty”, “a lot of difficulty” and “can’t see at all” columns in Table 2) was higher than that of females in the same category of visual difficulty, because the overall population surveyed included more males, the proportions of females who had difficulty was slightly higher than that of males with greater proportions reporting “some difficulty” (7.8% vs. 6.0%) and “a lot of difficulty” (2.3% vs. 1.3%). The Northern region has a higher proportion of visual difficulty, with 440 (9.6%) having “some difficulty” and 133 (2.9%) having “a lot of difficulty” with vision, while in the Central and Southern regions, visual difficulty was at relatively lower levels.

Among rural dwellers, 1171 (6.4%) had some visual difficulty, and 313 (1.7%) had a lot of visual difficulty. In the case of urban residents, a larger proportion, 380 (7.7%), had some visual difficulty, and only 83 (1.7) had “a lot of difficulty”. In terms of education level, the results show a clear gradient with a lot of visual difficulty reported among those with no education (3.5%). However, those who reported experiencing “some difficulty” were mostly individuals who completed higher education (10.3%). Visual difficulty was most common among widowed participants, with 350 (13.5%) reporting having some visual difficulty and 141 (5.5%) reporting having “a lot of difficulty”. Those who were never married had the lowest levels of impairment (91.2% reported no difficulty with vision).

### 3.3. Association Between Demographic Factors and Spectacle/Contact Lens Wear Among Persons Surveyed

There was a significant association between spectacle/contact lens wear and age, region of residence, education, and marital status (*p* < 0.001), but not the sex of the individuals surveyed (*p* = 0.125) (Table 3). The frequency of spectacle/contact lens wear increased with age, rising from 2.5% (15–19 years) to 12–14% in older age groups. In terms of regional distribution, participants in the Northern region reported the highest spectacle usage (8.0%) compared to 6.0% in other regions. Use of spectacle/contact lenses was also higher (10.4%) among urban residents compared to rural residents (5.3%). The use also increased with increasing educational level, reaching 19.0% among those with higher education compared to between 4.5% to 5.0% in participants with lower education levels. Widowed individuals had the highest usage of about 10.1%, while other marital groups had lower and similar levels of use. There was no significant difference by sex, with similar usage among males (6.6%) and females (6.1%).

### 3.4. Distribution of Spectacle/Contact Lens Wear Among Participants, Depending on Whether They Had Difficulty Seeing or Not

There was a strong association between spectacle/contact lens wear and visual difficulty (χ^2^ = 2110, *p* < 0.001) (Table 4). Among individuals with visual difficulties, the majority were not using spectacles or contact lenses, with approximately 67–70% of those reporting “some” or “a lot of difficulty” being uncorrected. Among those who don’t wear spectacles or contact lenses, 5% have some visual difficulty and 1.2% have “a lot of difficulty” (Table 4). Although not all visual difficulty in this survey may be directly attributed to refractive error, these results indicate a substantial unmet need for visual correction.

### 3.5. Multinomial Logistic Regression Showing Demographic Predictors of Visual Disability

Multinomial logistic regression (base category: “no difficulty” category, likelihood ratio test of the final model: χ^2^ = 2522.6, *p* < 0.001) showed that age, region and level of education were significant predictors of visual difficulty, while other factors showed more limited associations (Table 5). Overall, higher education, increasing age and regional differences were the strongest predictors of visual disability, with weaker and less consistent effects observed for sex, residence and marital status. Those with higher education were 3.5 times more likely to report some visual difficulties and were consistently high across subgroups. Increasing age was consistently associated with higher odds of reporting visual difficulty across all categories, including “some difficulty” (OR = 1.05), “a lot of difficulty” (OR = 1.07) and “cannot see at all” (OR = 1.08) (all *p* < 0.001). Region was also important, with participants from the Northern (OR = 1.8) and Central regions (OR = 1.4) having higher odds of reporting “some visual difficulty” (80% and 40% respectively). Sex, education, and marital status showed some association, particularly with the “some difficulty” category, while urban residence was only a weak predictor for reports of visual difficulty (OR: 1.1–1.2). Estimates for “cannot see at all” were unstable and likely unreliable due to the small sample sizes in these categories.

### 3.6. Multinomial Logistic Regression Showing the Association Between Demographic Variables and Spectacle/Contact Lens Wear

Overall, age, urban residence, and higher education were the strongest predictors of spectacle/contact lens wear (Table 6). Increasing age was significantly associated with higher odds of spectacle/contact lens wear (OR = 1.05, *p* < 0.001). Urban dwellers had a higher odds of using spectacles or contact lenses (OR = 1.5, *p* < 0.001). Education level also showed a gradient with individuals who completed secondary (OR: 1.9, *p* = 0.004) and especially higher education (OR = 5.0, *p* < 0.001) having increased odds of wearing spectacles/contact lenses, while those with no education were (50%) less likely to wear spectacles or contact lenses (OR = 0.5, *p* = 0.007). There were modest associations between participants’ sex and region (Northern region) and likelihood of spectacle or contact lens wear, while marital status was generally not associated, except for divorced/separated individuals, who were less likely to wear correction for visual difficulties (OR = 0.7, *p* = 0.010).

## 4. Discussion

The 2024 Malawi DHS demonstrates a substantial burden of visual difficulty among Malawians, with marked gradients across demographic and socioeconomic strata. The observed increase in visual difficulty with advancing age echoes patterns consistently reported in both global and regional literature [14,15]. In our analysis, the prevalence of “some visual difficulty” rose from 2.3% among younger individuals to 15.5% in those over 74, with parallel increases in more severe impairment. This trend plausibly reflects the cumulative impact of age-related ocular pathologies, notably cataract and uncorrected refractive error, conditions that remain leading causes of vision loss in sub-Saharan Africa [16]. It also links to the well-described growing global aging population and presence of presbyopia. It has been indicated that the uptake of refractive correction in this age group is only approximately 10% in LMICs [17].

Sex differences in this survey, in which women reported slightly higher rates of visual difficulty, are consistent with previous global studies, which reported women having a prevalence of 1.6% compared to men, 1.4% [16], and women accounting for 55% of all cases of visual impairment [15]. In SSA, a recent estimate [14] reported that women had a higher age-standardized prevalence than men for blindness (3.76% vs. 3.69%), moderate to severe visual impairment (12.17% vs. 11.53%), mild visual impairment (10.03% vs. 8.42%), and presbyopia (38.62% vs. 36.30%) among adults aged 50 years and older. While this may partly relate to underlying biological differences in disease prevalence, it more likely reflects persistent sex disparities in access to health services and health-seeking behaviors. In Malawi, males are reported to have a better eye care utilization behavior compared to females (OR 1.2, 95% CI 1.104–1.290) [18]. Notably, some studies have identified women as less likely to receive timely eye care, further compounding their risk of untreated impairment [18]. However, there was no association between sex and spectacle use in our data, suggesting progress toward sex equity or perhaps variation in cultural norms across regions in recent years.

Regional variation emerged as a notable theme, with the Northern region exhibiting higher odds of visual difficulty. This finding aligns with broader evidence that rural or less-resourced regions bear a disproportionate burden of avoidable blindness and visual impairment due to limited access to eye care services, infrastructure, and trained personnel [19,20,21]. The predominance of visual difficulty among rural dwellers, contrasted with higher spectacle use among urban residents, further highlights inequities in service provision, health literacy, and disposable income [22]. It is plausible that historical disparities in resource allocation, as well as sociocultural barriers, contribute to these persistent regional differences [23].

Socioeconomic and educational gradients in visual difficulty were also evident. Individuals with lower educational attainment experienced more severe visual impairment, a finding consistent with prior research linking low education to reduced health literacy and lower uptake of preventive and curative eye care [24,25]. Interestingly, those with higher education reported more “some difficulty,” which may reflect greater health awareness, differential symptom recognition, or increased likelihood of diagnosis and reporting [18,24]. Our finding that higher education is strongly associated with spectacle wear (OR = 5.0) echoes similar evidence from Ethiopia [26], Malawi [18] and Zimbabwe [27] that education influences eye care use, underscoring the role of education in facilitating access to and utilization of eye care services. Conversely, individuals with no education were 50% less likely to wear spectacles, highlighting the importance of targeted outreach and community education to bridge equity gaps. However, “reporting bias” should be considered when interpreting these patterns, especially in self-reported survey data [28].

Marital status played a significant role: widowed individuals bore the highest rates of visual difficulty and spectacle use. This may reflect a cumulative effect of aging and social disadvantage, as widowhood is often associated with older age and increased vulnerability [29]. Social isolation or diminished family support may further impede access to eye care and rehabilitation services for this group.

Despite considerable self-reported visual difficulty, the uptake of spectacles or contact lenses remains strikingly low, with only 6–7% overall, rising modestly to 19% among the most educated respondents. Most individuals with visual difficulty (67–70%) remained uncorrected, outlining a substantial unmet need for refractive services. Comparable levels of unmet need have been documented across sub-Saharan Africa, with high unmet eye health needs, with only 12% receiving glasses to address blurred vision [30]. Whilst lower eye care utilization rates of between 30–58% have been reported in developed countries [31]. Our findings reinforce the urgency of closing this gap to avert avoidable vision loss and abate the recent improvement in the region [8].

Barriers to optical correction in Malawi likely include the high cost, limited availability of optical services, low awareness due to communication difficulties, and cultural beliefs, as documented in SSA [20,21]. Addressing only supply-side constraints is insufficient; the Ministry of Health, Education, communities and development partners should collaborate to implement demand-side interventions, such as health education and community mobilization, which are critical for improving uptake, particularly among marginalized groups. Given Malawi’s predominantly rural population and the high prevalence of uncorrected refractive error in low- and middle-income countries [32], scaling up community-based vision screening, integrating eye health into primary care, and addressing cost and supply chain barriers for spectacles are crucial interventions [22,32]. However, implementation research is needed to evaluate the feasibility and impact of these strategies in diverse Malawian contexts.

Several limitations warrant consideration. The reliance on self-reported data may lead to misclassification or underestimation of the true prevalence, particularly in populations with low health literacy or limited symptom awareness. Additionally, small sample sizes in severe impairment groups constrain the precision of subgroup analyses. Future studies should consider objective vision assessments and longitudinal designs with adequate representation in all vision disability categories to enhance causal inference and trend monitoring, and consider technology-enabled interventions to support refractive service coverage through decentralized prevention and screening that will improve access for vulnerable populations.

## 5. Conclusions

The 2024 Malawi DHS findings highlight an urgent need for enhanced eye care services, especially for older adults, rural residents, and individuals with lower levels of education. Tackling the high unmet need for refractive correction through affordable, accessible services, strengthened public education, and targeted outreach like community-based vision screening programs, mobile eye services to underserved communities, offering subsidies or national health insurance for eye care services to the financially marginalized, could substantially reduce the burden of avoidable visual impairment in Malawi. Future research should explore innovative models of service delivery such as mobile eye care and tele-optometry and/or ophthalmology, strategies to improve equity in access, and interventions to address both supply and demand-side barriers.

## Figures and Tables

**Table 1:** Demographic distribution of individuals surveyed.

Variables	Categories	Frequency (Percentage)
Age	15–19	399 (1.7)
	20–24	2231 (9.7)
	25–29	2876 (12.5)
	30–34	2885 (12.5)
	35–39	2877 (12.5)
	40–44	2521 (10.9)
	45–49	2061 (8.9)
	50–54	1894 (8.2)
	55–59	1314 (5.7)
	60–64	1277 (5.5)
	65–69	853 (3.7)
	70–74	770 (3.3)
	>74	1137 (4.9)
Sex	Male	14,385 (62.3)
	Female	8710 (37.7)
Region	Northern	4577 (19.8)
	Central	7958 (34.5)
	Southern	10,560 (45.7)
Type of residence	Urban	4928 (21.3)
	Rural	18,167 (78.7)
Highest education level attained	No education, preschool/early childhood	2738 (11.9)
	Primary	12,866 (55.7)
	Secondary	5902 (25.6)
	Higher	1288 (5.6)
	Don’t know	301 (1.3)
Marital status	Married or living together	15,830 (68.5)
	Divorced/separated	3632 (15.7)
	Widowed	2575 (11.2)
	Never married or lived together	1058 (4.6)

**Table 2:** Association between demographic factors and visual difficulty among the population surveyed.

Variables	Categories	Visual Disability Category: Frequency (%)					Pearson Chi Square—χ^2^ (Cramer’s V)	*p*-Value
		No Difficulty (%)	Some Difficulty (%)	A Lot of Difficulty	Can’t See at All	Don’t Know		
Age	15–19	383 (96.0)	9 (2.3)	0 (0.0)	0 (0.0)	7 (1.8)	2474.4 (0.2)	**<** **0** **.001**
	20–24	2140 (95.9)	51 (2.3)	7 (0.3)	0 (0.0)	33 (1.5)		
	25–29	2730 (94.9	81 (2.8)	10 (0.3)	0 (0.0)	55 (1.9)		
	30–34	2705 (93.8)	90 (3.1)	21 (0.7)	0 (0.0)	69 (2.4)		
	35–39	2692 (93.6)	89 (3.1)	16 (0.6)	1 (0.0)	79 (2.7)		
	40–44	2237 (88.7)	143 (5.7)	22 (0.9)	1 (0.0)	118 (4.7)		
	45–49	1713 (83.1)	179 (8.7)	26 (1.3)	2 (0.1)	141 (6.8)		
	50–54	1505 (79.5)	188 (9.9)	37 (2.0)	2 (0.1)	162 (8.6)		
	55–59	1015 (77.2)	149 (11.3)	37 (2.8)	1 (0.1)	112 (8.5)		
	60–64	937 (73.4)	169 (13.2)	40 (3.1)	4 (0.3)	127 (9.9)		
	65–69	595 (69.8)	108 (12.7)	35 (4.1)	3 (0.4)	112 (13.1)		
	70–74	495 (64.3)	119 (15.5)	46 (6.0)	2 (0.3)	108 (14.0)		
	>74	646 (56.8)	176 (15.5)	99 (8.7)	7 (0.6)	209 (18.4)		
Sex	Male	12,644 (87.9)	869 (6.0)	194 (1.3)	13 (0.1)	665 (4.6)	164.1 (0.1)	**<** **0** **.001**
	Female	7149 (82.1)	682 (7.8)	202 (2.3)	10 (0.1)	667 (7.7)		
Region	Northern	3772 (82.4)	440 (9.6)	133 (2.9)	4 (0.1)	228 (5.0)	208.7 (0.1)	**<** **0** **.001**
	Central	6915 (86.9)	541 (6.8)	139 (1.7)	8 (0.1)	355 (4.5)		
	Southern	9106 (86.2)	570 (5.4)	124 (1.2)	11 (0.1)	749 (7.1)		
Type of residence	Urban	4249 (86.2)	380 (7.7)	83 (1.7)	0 (0.0)	216 (4.4)	36.5 (0.04)	**<** **0** **.001**
	Rural	15,544 (85.6)	1171 (6.4)	313 (1.7)	23 (0.1)	1116 (6.1)		
Highest Education level	No education, preschool/ early childhood	2179 (79.6)	217 (7.9)	97 (3.5)	3 (0.1)	242 (8.8)	256.7 (0.1)	**<** **0** **.001**
	Primary	10,962 (85.2)	866 (6.7)	195 (1.5)	17 (0.1)	826 (6.4)		
	Secondary	5291 (89.6)	319 (5.4)	82 (1.4)	2 (0.0)	208 (3.5)		
	Higher	1096 (85.1)	133 (10.3)	17 (1.3)	1 (0.1)	41 (3.2)		
	Don’t know	265 (88.0)	16 (5.3)	5 (1.7)	0 (0.0)	15 (5.0)		
Marital status	Married or living together	13,980 (88.3)	918 (5.8)	190 (1.2)	12 (0.1)	730 (4.6)	816.3 (0.1)	**<** **0** **.001**
	Divorced/separated	3087 (85.0)	228 (6.3)	58 (1.6)	4 (0.1)	255 (7.0)		
	Widowed	1761 (68.4)	350 (13.6)	141 (5.5)	7 (0.3)	316 (12.3)		
	Never married or lived together	965 (91.2)	55 (5.2)	7 (0.7)	0 (0.0)	31 (2.9)		

*NB: Bolded p-value means statistically significant association; Percentages are calculated based on row totals.*

**Table 3:** Association between demographic factors and spectacle/contact lens wear among persons surveyed.

Variables	Categories	Wear Spectacles/Contact Lenses		Pearson Chi Square—χ^2^ (Cramer’s V)	*p*-Value
		No (%)	Yes (%)		
Age groups	15–19	389 (97.5)	10 (2.5)	658.9 (0.2)	**<0.001**
	20–24	2186 (98.0)	45 (2.0)		
	25–29	2793 (97.1)	83 (2.9)		
	30–34	2779 (96.3)	106 (3.7)		
	35–39	2776 (96.5)	101 (3.5)		
	40–44	2404 (95.4)	117 (4.6)		
	45–49	1930 (93.6)	131 (6.4)		
	50–54	1703 (89.9)	191 (10.1)		
	55–59	1151 (87.6)	163 (12.4)		
	60–64	1106 (86.6)	171 (13.4)		
	65–69	746 (87.5)	107 (12.5)		
	70–74	660 (85.7)	110 (14.3)		
	>74	996 (87.6)	141 (12.4)		
Sex	Male	13,438 (93.4)	947 (6.6)	2.4 (0.01)	0.125
	Female	8181 (93.9)	529 (6.1)		
Region	Northern	4210 (92.0)	367 (8.0)	25.3 (0.03)	**<0.001**
	Central	7478 (94.0)	480 (6.0)		
	Southern	9931 (94.0)	629 (6.0)		
Type of residence	Urban	4414 (89.6)	514 (10.4)	170.9 (0.1)	**<0.001**
	Rural	17,205 (94.7)	962 (5.3)		
Highest Education level	No education, preschool/early childhood	2614 (95.5)	124 (4.5)	411.1 (0.1)	**<0.001**
	Primary	12,220 (95)	646 (5.0)		
	Secondary	5465 (92.6)	437 (7.4)		
	Higher	1043 (81.0)	245 (19.0)		
	Don’t know	277 (92.0)	24 (8.0)		
Marital status	Married or living together	14,837 (93.7)	993 (6.3)	88.1 (0.1)	**<0.001**
	Divorced/separated	3477 (95.7)	155 (4.3)		
	Widowed	2314 (89.9)	261 (10.1)		
	Never married or lived together	991 (93.7)	67 (6.3)		

*NB: Bolded p-value means statistically significant association; Percentages are calculated based on row totals.*

**Table 4:** Distribution of spectacle/contact lens wear among participants, depending on whether they had difficulty seeing or not.

Wear Spectacle or Contact Lens		Visual Difficulty Categories					Total (%)	Chi Square—χ^2^ (Cramer’s V)	*p*-Value
		No Difficulty (%)	Some Difficulty (%)	A Lot of Difficulty (%)	Can’t See at All (%)	Don’t Know (%)			
	Yes	791 (53.6)	464 (31.4)	131 (8.9)	6 (0.4)	84 (5.7)	1476 (100)	2110 (0.3)	**<0.001**
	No	19,002 (87.9)	1087 (5.0)	265 (1.2)	17 (0.1)	1248 (5.8)	21,619 (100)		

*NB: Bolded p-value means statistically significant association.*

**Table 5:** Multinomial Logistic regression showing demographic predictors of visual disability.

Predictors	Some Difficulty OR (95% CI)	*p*-Value	A Lot of Difficulty OR (95% CI)	*p*-Value	Cannot See at All OR (95% CI)	*p*-Value	Don’t Know OR (95% CI)	*p*-Value
Age	1.05 (1.04–1.1)	**<0.001**	1.067 (1.06–1.074)	**<0.001**	1.08 (1.06–1.1)	**<0.001**	1.05 (1.048–1.06)	**<0.001**
Sex	0.8 (0.7–0.9)	**0.003**	0.9 (0.6–1.2)	**0.03**	1.1 (0.3–3.9)	0.869	0.7 (0.6–0.8)	**<0.001**
Northern Region	1.8 (1.6–2.0)	**<0.001**	2.7 (2.1–3.5)	**<0.001**	0.8 (0.3–2.6)	0.754	0.7 (0.6–0.9)	**<0.001**
Central Region	1.4 (1.2–1.5)	**<0.001**	1.7 (1.3–2.2)	**<0.001**	1.0 (0.4–2.6)	0.944	0.7 (0.6–0.8)	**<0.001**
Urban Residence	1.2 (1.0–1.3)	**0.026**	1.1 (0.8–1.4)	0.527	3.1 × 10^−8^ (10^−10^–0.0) *	0.994	0.9 (0.7–1.0)	0.082
No Education	1.3 (0.8–2.3)	0.306	1.7 (0.7–4.4)	0.248	1.2 × 10^7^ (0.0–10^10^) *	0.998	1.4 (0.8–2.5)	0.201
Primary Education	1.8 (1.1–3.1)	**0.024**	1.5 (0.6–3.7)	0.403	3.7 × 10^7^ (0.0–10^10^) *	0.998	2.0 (1.2–3.5)	**0.011**
Secondary education	1.9 (1.1–3.3)	**0.018**	2.3 (0.9–5.9)	0.086	2.7 × 10^7^ (0.0–10^10^) *	0.998	1.7 (1.0–3.0)	0.068
Higher Education	3.5 (2.0–6.0)	**<0.001**	2.1 (0.8–6.0)	0.156	9.8 × 10^7^ (0.0–10^10^) *	0.998	1.5 (0.8–2.8)	0.215
Married/Living together	0.7 (0.5–0.9)	**0.009**	0.7 (0.3–1.5)	0.329	4.2 × 10^6^ (1.0 × 10^6^–1.7 × 10^6^) *	**<0.001**	0.7 (0.4–1.0)	**0.028**
Divorced/separated	0.8 (0.5–1.0)	0.105	1.1 (0.47–2.4)	0.879	8.6 × 10^6^ (2.3 × 10^6^–3.2 × 10^7^) *	**<0.001**	0.9 (0.6–1.3)	0.466
Widowed	0.9 (0.6–1.2)	0.417	1.3 (0.6–3.0)	0.523	7.1 × 10^6^ (7.0 × 10^6^–7.2 × 10^7^) *	-	0.7 (0.5–1.1)	0.126

NB: Bolded p-value means statistically significant association The “don’t know” columns are included to ensure completeness of reporting. * Note: Due to a small sample size in these cells, the confidence interval is likely extremely wide; hence, the estimate is unreliable. The categories not shown represent the reference categories.

**Table 6:** Multinomial Logistic regression showing the association between demographic variables and spectacle/contact lens wear.

Predictors	Odds Ratio (OR)	95% Confidence Interval (CI)		
		Lower Bound	Upper Bound	*p*-Value
Age	1.047	1.043	1.05	**<0.001**
Sex	0.9	0.7	1.0	**0.041**
Northern Region	1.2	1.0	1.4	**0.016**
Central Region	1.1	1.0	1.2	0.148
Urban Residence	1.5	1.3	1.7	**<0.001**
No Education	0.5	0.3	0.8	**0.007**
Primary Education	1.0	0.6	1.5	0.941
Secondary education	1.9	1.2	3.1	**0.004**
Higher Education	5.0	3.1	8.0	**<0.001**
Married/Living together	0.9	0.6	1.1	0.288
Divorced/separated	0.7	0.5	0.9	**0.010**
Widowed	0.8	0.6	1.1	0.201

*NB: Bolded p-value means statistically significant association.*

## Data Availability

Data can be downloaded from the official DHS website: http://www.dhsprogram.com (accessed on 21 April 2026).
